# Development of a New Dislodgeable Foliar Residue Analytical Laboratory Method for Pesticides

**DOI:** 10.1093/annweh/wxac045

**Published:** 2022-06-29

**Authors:** Mohamed H Badawy, Darragh Murnane, Kathleen A Lewis, Neil Morgan

**Affiliations:** The School of Life and Medical Science, University of Hertfordshire, School of Life and Medical Sciences, Hatfield, Herts AL10 9AB, UK; The School of Life and Medical Science, University of Hertfordshire, School of Life and Medical Sciences, Hatfield, Herts AL10 9AB, UK; The School of Life and Medical Science, University of Hertfordshire, School of Life and Medical Sciences, Hatfield, Herts AL10 9AB, UK; Product Safety Department, Syngenta, Jealott’s Hill International Research Centre, Bracknell, Berkshire RG42 6EY, UK

**Keywords:** DFR, dislodgeable foliar residue, non-dietary risk assessment, pesticide residue analysis, pesticide risk assessment, pesticides, worker exposure

## Abstract

The dislodgeable foliar residue (DFR) is the amount of pesticide that exists on foliage after the pesticide has dried and which could dislodge to the skin or clothes of workers and is a key parameter for non-dietary risk assessments required to demonstrate safe use for pesticide registration. DFR data in the literature are described as insufficiently reliable, limited, and encompasses considerable statistical uncertainties. The purpose of this article is to describe a newly developed laboratory method for the quantification of DFR with an illustrative example. The laboratory method reflected available field DFR methodology but involved controlled application of droplets to leaves and validation of the wash-off process used to remove the residue from the leaf surface before the analytical quantification. A very high level of accuracy (99.7–102.1%) and precision (±1.5%) was achieved. Residue data generated from the illustrated application of the method showed a robust normal distribution, unlike field studies. The method is deemed to be controllable, cost-efficient, and time-saving, taking hours rather than days. This enables the generation of more data to allow extrapolation between the generated data by investigating multiple factors that may influence DFR. An improved understanding of DFR could save time, money, and resources.

**What’s important about this paper**

Pesticide residue on foliage can pose a risk to workers and others that enter fields after application and is an important component of pesticide risk assessment. This study demonstrates a new laboratory method for measuring dislodgeable foliar pesticide residue (DFR) that is fast, easy, and cost-effective for data generation. The laboratory method will allow factors that may influence DFR to be studied under controlled conditions, and can be used with in-field data to refine worker risk assessments in the future.

## Introduction

Pesticide use is determined by regulatory agencies worldwide to ensure the proper, safe, and consistent use of pesticides. Accordingly, a pesticide risk assessment is considered an important component of pesticide regulation in most of the developed world (Krieger and Ross, 1993). In the European Union (EU), as published in the most recent European Food Safety Authority (EFSA) guidance for Regulation (EC) No. 1107/2009, the risk assessment for plant protection products (PPPs) must be carried out for all exposure scenarios for operators, workers, residents, and bystanders that can be expected to occur as a consequence of the proposed uses of PPPs. Most of these scenarios will fall into a category for which standardized first-tier exposure assessment can be carried out according to the guidance, using previously set default input values. For those scenarios that are not covered in or do not satisfy the first-tier assessment, an *ad hoc*, higher tier exposure assessment may also be used by the applicant by generating experimental data based on actual exposure (EFSA, 2014a; Charistou *et al.*, 2022).

The outcomes of the public consultations on the guidance also identified various scenarios for which exposure estimates were least satisfactory due to data gaps, and recommendations were made for further research that would reduce current uncertainties (EFSA, 2014b; Charistou *et al.*, 2022). Despite the new data generated by Baumann *et al.* (2019) attempting to address the uncertainties, and included in the new EFSA guidance, the available data and guidance remain insufficient and limited (Baumann *et al.*, 2019; Charistou *et al.*, 2022). From EFSA guidance 2014 and 2022, it is clear that the available data for worker exposure are not reliable enough due to the limited data sets and statistical uncertainties that exist (EFSA, 2014a; Charistou *et al.*, 2022). Hence, the *ad hoc* EFSA working group (‘WoG’) strongly recommended further collection and production of data on specific Transfer Coefficients (TC) and dislodgeable foliar pesticide residue (DFR) values to produce more realistic exposure assessments (EFSA, 2014b; Charistou *et al.*, 2022). During re-entry activities, the most important route of exposure is dermal. The level of resultant exposure for any activity depends on the amount of DFR, the intensity of contact with the foliage, and the duration of the contact (EFSA, 2014a). In the absence of experimental DFR data, the default value of 3 µg (i.e. 3 µg active substance cm^−2^ of foliage)/(kg a.s. applied ha^−1^) should be used, unless measured DFR data are needed to demonstrate a safe use, when a DFR study must be conducted. Nevertheless, such value is regarded as highly conservative (Lewis and Tzilivakis, 2017; Charistou *et al.*, 2022).

Historically, the DFR determination method was first developed by Gunther *et al.* (1973) 49 years ago. In 2009, the method was then published by the USA Environmental Protection Agency (EPA) in the Occupational and Residential Exposure Test Guidelines (OPPTS 875.2100 Foliar Dislodgeable Residue Dissipation) (EPA, 2009). The same method is also recommended in the DFR USA Agriculture Task Force (ARTF) draft protocol and has been broadly used in the open literature (Kasiotis *et al.*, 2017; Charistou *et al.*, 2022). The EPA Guidelines provide a description of the technique and sampling methods used to quantify DFR which is based on the definition, ‘*DFRs are the amount of chemical residues deposited onto the leaf surface that has not been absorbed into the leaf or dissipated from the surface, and that can be dislodged by shaking leaf samples in a detergent solution*’ (Gunther *et al.*, 1973). The guideline is intended to meet testing requirements of the Federal Insecticide, Fungicide, and Rodenticide Act (FIFRA) according to the EPA Test Guidelines for Pesticides and Toxic Substances Uses (EPA, 2009). The test guidelines (OPPTS 875.2100) are referenced by the European Commission (EC) in a document on the authorization of PPPs in Europe (European Commission, 2020) which mandated the study in Europe to follow the Good Laboratory Practice standards (GLP). Despite the presence of the OPPTS Guidelines 875.2000 and 875.2100, there is still no harmonized method for conducting DFR studies throughout the open literature and most of the current studies follow the previously mentioned method with minor differences (BfR, 2020).

The process of method validation for any experiment is crucial for the method’s reproducibility and accuracy. Several validation attempts for the DFR method have been made by Bruce *et al.* (2006). These attempts studied the effect of different types of washing solutions, different washing volumes, wash duration, and shaking methods that were used in some of the DFR studies in the literature to compare them with the EPA-developed method (OPPTS 875.2100 Foliar Dislodgeable Residue Dissipation) (EPA, 2009). This developed DFR method involved soaking 400 cm^2^ of a total leaf surface area in two single washes of 100 ml of Aerosol OT 0.01% (bis(2-ethylhexyl) sulfosuccinate, sodium salt dioctyl sodium sulfosuccinate) for 10 min on a reciprocating shaker (EPA, 2009). Notwithstanding these validation attempts were on a very small scale and involved only two pesticides and two leafy crops (i.e. cabbage and lettuce), they did not reveal statistically different results between most of the employed techniques except for one when compared with the EPA method (Bruce *et al.*, 2006). The variability that exists among DFR field studies is often attributed to the seasonal nature of such studies, which usually encompasses non-controllable effects, such as changes in the metrological conditions. Besides different techniques, units, and formulations used in the available DFR studies, a direct comparison between these studies is not possible. This has been highlighted in the latest EFSA guidance on the assessment of exposure of operators, workers, residents, and bystanders in the risk assessment of PPPs (Charistou *et al.*, 2022).

The DFR definition, however, considers that all pesticides that exist on the surface after the pesticides dry are dislodgeable, and therefore, ensuring that no pesticide residue is left on the leaf was crucial to conclude a precise DFR from the current lab method (EFSA, 2014a). Moreover, according to the only available guideline for testing the DFR ‘the EPA Occupational and Residential Exposure Test Guidelines OPPTS 875.2100 Foliar Dislodgeable Residue Dissipation [EPA 712–C–96–267]’ (EPA, 2009), DFRs represent chemical residues on the surfaces of treated foliage that are available for transfer to exposed populations (e.g. re-entry workers) during contact with those treated leaf surfaces. It, therefore, follows, that from the EPA (2009) guidelines, the definition of DFR requires all residues found on the surface after the pesticide dry considered dislodgeable and should be quantified during the dislodging procedures, and therefore extraction-to-exhaustion was crucial in the current laboratory method.

The initial guidelines of the current method did not include any validation of the wash-off solution used and its efficiency in dislodging all the residue from the plant surface. Moreover, the method guidelines emphasized the need for further validation requirements on the efficiency of the dislodging procedure (EPA, 2009). That highlights the importance of validating the volume of wash-off solution needed for each crop or leaf type before conducting the DFR field studies required by the non-dietary risk assessment of the PPPs (EFSA, 2014b).

Monitoring of pesticide residue on plants is considered the gold standard to evaluate pesticide safety. Although generating enough data through conducting DFR field experiments is a robust way to derive a more realistic DFR default value for regulatory non-dietary risk assessment, it is not always achievable. The reason for this is not only because field experiments are generally expensive, seasonal, and time-consuming (BfR, 2020) but also because of the privacy and ownership of the generated data across the biggest agrochemical companies in the industry. Hence, this study aimed to develop a new standardized laboratory method for quantifying DFR for research purposes with an application example and description of the technique as a proof of concept. This newly introduced method could be an important asset to generate sufficient DFR data on many targeted crops under controlled and manageable environmental conditions so that the data could be used in conjunction with the existing experimental data for the regulatory authorities to set accurate and more reflective DFR default values for various crop groups and pesticides. Furthermore, as the proposed technique is relatively rapid, it would also allow investigating multiple factors that could influence DFR and facilitate the future validation of DFR assessment of multiple plant types and formulations. The laboratory DFR method would potentially allow further extrapolation between DFR studies if any correlation among the influencing factors is proven. This eventually would save time, money, and resources for both the industry and the registration authorities.

## Materials and methods

The description of the DFR analytical method below is illustrated with a practical example. The technique involved testing different leaves from different crops: French bean, tomato, soybean, oilseed rape, and wheat. These crops were selected with variabilities among them in their leaf architecture/structure besides their fast and easy growing conditions required.

All crop leaves were tested on one exemplar formulation of 10% difenoconazole (DFZ). The formulation tested was an emulsifiable concentrate (EC) which is ‘A liquid, homogenous preparation to be applied as an emulsion after dilution in water’ (CIPAC, 2016). DFZ EC 10% formulation was formulated at Syngenta Jealott’s Hill International Research Station for research purposes only. This formulation is not registered nor commercially used. The previously mentioned crops were selected for the estimation and validation of the new technique. The application of this method is not exclusively limited to the above-mentioned pesticide nor the crop types and could be used for testing any crop/pesticide combinations.

### Plant growth and selection

**Figure 1.**
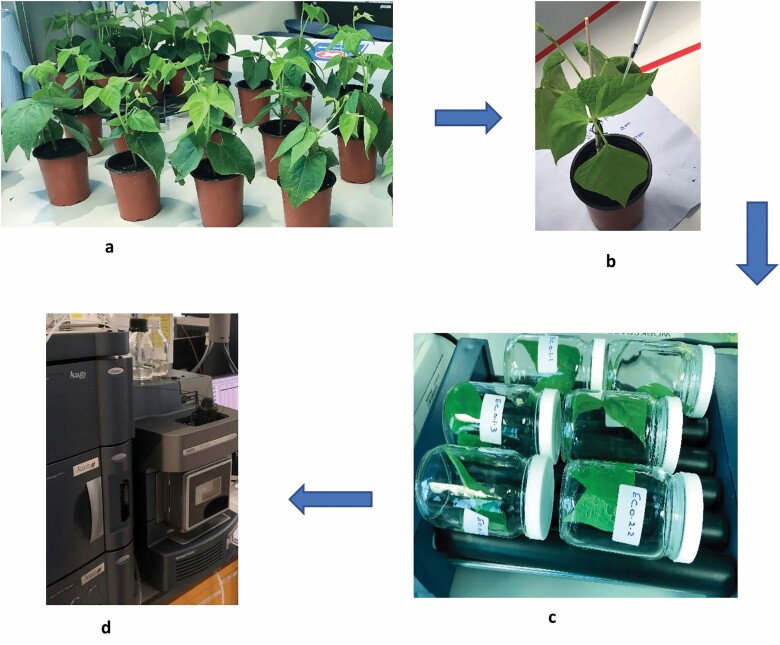
Descriptive summary of the DFR laboratory methodology. This figure elucidates with pictures the new laboratory technique used to evaluate the DFR of pesticides. (a) Plants were sown in all-purpose compost in Sanyo growth chamber. (b) Plant leaves were treated with an electronic micropipette generating a uniform droplet of 0.2 µl. (c) The glass bottles were left rolling on an electronic roller for a complete rinse of the residue from the plant surface using Aerosol OT 0.01% (w/v). (d) DFR chromatographic analysis was carried out using a LC–MS.

Plants were grown in all-purpose commercially available compost. A Sanyo versatile environmental growth chamber Model MLR-351 purchased from SANYO Electric Company, Sussex, UK was used for growing the plants as shown in Fig. 1. The Sanyo chamber was adjusted to provide optimum growing conditions for the uniform growth of each plant as per the optimum conditions mentioned in Table 1. All plants were watered uniformly through the capillary matting system (3 mm thickness) underneath the pots in plastic trays to preserve the soil moisture. All plants were kept in the growing chamber throughout their growing period. Before treatment, plants with an approximately similar growth stage, height, and leaf size were selected to minimize the variabilities among the plants in the experiment as well as ensure that selected plants were free from any infestation and deformities. Approximate plant heights (stems) were measured using a ruler from the soil surface to the tip of the plant. Approximate leaf surface areas were measured using the millimetre graph paper method by taking a leaf, tracing it over the paper, and counting the squares covered by the leaf to give the area of one surface which is then multiplied by two to estimate the double-sided surface area of the leaf (Fascella *et al.*, 2009).

**Table 1.** Optimum growing condition and stage for plants selected for the DFR lab method.

Plant’s type (variety)	Sowing depth (cm)	Temperature (°C)		Light duration (h)		Targeted and selected leaf	^*a*^ Surface area (two leaves double sided) (cm^2^)	Plant height (cm)
		Day	Night	Day	Night			
Dwarf French bean ‘*Phaseolus vulgaris*’ Variety: Tendergreen	3–4	25	20	16	8	Both lateral leaves of the second trifoliate emerged	45	13
Wheat ‘*Triticum aestivum*’ Variety: Skyfall	2–3	20	16	14	10	First and second emerged true leaf	28	12
Oilseed rape ‘*Brassica napus*’ Variety: Charger	2–3	20	16	14	10	Second and third emerged leaves	132	7
Tomato ‘*Solanum lycopersicum*’ Variety: Alicante	0.5–1	25	20	16	8	Three leaves on the tip of the Second emerged branch	58	12
Edamame Soya Bean ‘*Glycine max*’ Variety: Green Shell	3–4	25	20	16	8	Both lateral leaves of the second emerged triflate	65	17

This table describes the optimum conditions set for sowing and growing selected plants to test both the DFR laboratory method and potential factors that may affect DFR.

^*a*^Approximate leaves surface area was measured using the millimetre graph paper method (Fascella *et al.*, 2009b).

### Pesticide spray and washing solution preparation

- Pesticide spray preparation

The pesticide was prepared fresh on the treatment day by diluting the pesticide formulation in distilled water to match field dilution according to the Good Agriculture Practice (GAP) standards for DFZ EC 10%. The prepared concentration (approximately 0.625 mg ml^−1^) corresponds to the average field application rate for French beans (125-g DFZ per 200 l per ha).

- Preparation of the leaf washing solution

Residues were dislodged from plant leaves following OPPTS Guidelines and prior reports (Iwata *et al.*, 1977; EPA, 2009) using an aqueous surfactant solution of Aerosol OT 0.01% (w/v) (from ThermoFisher Scientific, Stortford, UK). As the shelf life of the Aerosol OT 0.01% (w/v) at room temperature is 48 h, a stock of 0.01% (w/v) concentration was prepared fresh on the treatment day.

### Plant treatment

Before treating the plants for estimating the residue on the surface (DFR), validating the appropriate amount of solution for efficient wash-off was crucial to ensure the dislodgeable fraction of the residue is thoroughly rinsed off and recovered from the treated leaves. The current approach involved rinsing of the entire leaf, in contrast to alternative techniques such as leaf punching that can only sample a limited surface area of pesticide application. Five replicates of two leaves of each crop mentioned in Table 1 were selected for the validation process.

Treatment was performed by dispensing 40 uniform droplets (20 droplets per leaf) of 0.2 µl each onto the surface of the targeted leaves (2 leaves for each replicate). The plants were treated with DFZ 10% EC prepared with a concentration of approximately 0.625 mg ml^−1^. Picus® Electronic Pipette, Single Channel model 735021 purchased from Sartorius Lab Instruments GmbH & Co. KG, Goettingen, Germany, was used. The electronic micropipette was used to generate droplets of a 0.2 µl size, the smallest reproducible volume that any commercially available micropipette can achieve; to mimic the actual spray in the field. Thus, the amount of DFZ that is expected to be on each tested replicate (2 leaves) is 5 µg. Plants were kept stable at room temperature (22°C) for 3 h after the treatment allowing the sprayed pesticide to dry on the leaf surface before cutting the treated leaves at the petiole using clean scissors and forceps. Leaves were then placed in clean and securely capped glass bottles before being washed in multiple, consecutive 15 ml volumes of freshly prepared Aerosol OT 0.01% (w/v). The glass bottles were left rolling on an electronic roller for 15 min before the solution was removed and replaced with a fresh aliquot of 15 ml washing solution. This was repeated a further two intervals, such that every two leaves were washed with at least three aliquots of 15 ml for a total of 45 min. Before the chromatographic analysis, decanted residue solutions for each wash were labelled and stored at (−4°C) in a cold room. The residue analysis was done within a week of the storage.

### Chromatographic analysis

A Waters Corp. (Manchester, UK) Xevo TQ-S tandem quadrupole mass spectrometer coupled to a Waters Acquity UPLC I-Class was used for HILIC-MS/MS analysis. The chromatography was conducted using a 1.8 µm, 2.1 × 50 mm Waters Acquity carbon 18UPLC column (Waters Limited, Wilmslow, UK). The equilibrium time for the column was 0.1 min and the column was held at 40°C (±5) while the sample injection volume was 1 µl. Mobile phase (A1) consisted of OPTIMA grade water with 0.2% formic acid and mobile phase (B1) was 100% Acetonitrile OPTIMA grade. The analysis included two transitions from the Q1 molecular ion (MH^+^) 406.085 *m*/*z* to Q3 111.0396 *m*/*z* with confirmatory Q3 of 251.0702 *m*/*z*, ES+ (DFZ) using the mass spectrometer for the primary and confirmatory detection of DFZ, respectively. The total mobile phase flow rate was 0.4 ml min^−1^ throughout the analysis. The mobile phase gradient started with A1 (40%): B1 (60%) for 1 min followed by a continuous increase in the organic solvent flow to A1 (20%): B1 (80%), A1 (10%): B1 (90%) at 3 and 3.10 and up to 4 min, respectively, before being returned to the starting condition of A1 (40%): B1(60%) until the end of the run. The running time was 7 min and the retention time of the DFZ was approximately 1.14 min.

Liquid chromatography–mass spectrophotometer (LC–MS) chromatogram of the blanks and sample solution validation was visually examined to ensure the integrity of the analysis and the existence of any signal interference. The limit of detection (LOD) and limit of quantification (LOQ) were set by assessing the precision and accuracy levels of a concentration gradient ranging from 0.0002 and up to 0.08 µg ml^−1^ along with the determination of minimum concentration with a noise-to-peak ratio greater than 10:1. Linearity was assessed as a part of the analytical method validation where a linear response was observed for two sets of DFZ calibration standards at five concentration levels by dilution using 50:50 of 100% Acetonitrile and 0.01% Aerosol OT in the range 0.004–0.04 µg ml^−1^. The concentration gradient for the two sets were prepared from the 98.8% DFZ analytical standard provided and manufactured by Syngenta Jealott’s Hill International Research Station, UK for research purposes.

Five fortification levels of DFZ equivalent to 0.2, 0.1, 0.08, 0.04, and 0.016 µg ml^−1^ (two replicates at each level) were analysed as part of the quality control procedure. The accuracy and precision of the validation method were performed according to the SANTE/2020/12830 Rev.1 methods (European Commission, 2021) where a minimum of two fortification levels appropriate to the LOQ, and the likely residue level or 10× LOQ should be assessed with an ideal mean recovery range from 80 to 110% (European Commission, 2021). For precision, the relative standard deviation (RSD%) should be ≤2% per fortification level. Seven replicates were used for each concentration level tested for accuracy and precision using the regression equation calculated from the regression function ‘*y* = *bx* + *a*’ where *b* value is the slope coefficient, *a* is the intercept constant, *y* is the dependent variable (plotted on the *y*-axis), and *x* is the independent variable (plotted on the *x*-axis). Samples were prepared in a final volume of 1 ml by diluting 0.5 ml of the residue in the washing solution with 0.5 ml of Acetonitrile 100% LC–MS grade.

### Method application exemplar: estimation of DFZ EC 10% DFR on oilseed rape leaves

Following the development of the method above, an example of the estimation of the DFZ EC 10% DFR on oilseed rape leaves was conducted for illustrative purposes. Ten replicates representing pots of the grown plant to the stage of the fourth emerged leaf were treated with freshly prepared DFZ 10% EC with a concentration of 0.625 mg ml^−1^ by dispensing 40 droplets (each of 0.2 µl size) using the electronic micropipette onto both the second and third emerged leaf from each potted plant. This was followed by both the leaf washing procedure and the quantification of dislodged residues using the LC–MS method as described above. A control group with untreated plants was also included in the experiment to identify any assay interference from the untreated plant leaves.

### Statistical analysis

Descriptive statistical analysis and the normality test (Shapiro–Wilk test) have been used to describe the DFR data of DFZ EC 10% sprayed on the oilseed rape plant. The descriptive analysis has been performed using SPSS, IBM version 27.0.

## Results

### Analytical validation of DFZ

The DFZ analysis followed a precision and accuracy level higher than the levels accepted in the referenced method SANTE/2020/12830 Rev.1 methods (European Commission, 2021) by following an acceptable range of precision (RSD ≤98%) and accuracy (RSD ≤2%). The practical LOQ was set to 0.004 µg ml^−1^ while the LOD level was set to 0.002 µg ml^−1^ for the DFZ analysis as per the levels of precision and accuracy (see Supplementary Material, available at *Annals of Work Exposures and Health* online). The calibration curve of the DFZ analysis proved linearity with a correlation coefficient *R* > 0.999 and the regression equation generated was *Y* = 2*E*+06*X* + 710.02 which was used to interpret all the concentrations of the analysis based on the LC–MS response. From the visual identification of the determined DFZ chromatographic peak at the LOQ, there was no interference from co-eluting peaks indicated at the retention time of DFZ. The mean recovery of each sample solution of the five fortification levels of DFZ EC 10% (0.21, 0.1, 0.08, 0.04, and 0.016 µg ml^−1^) were all within the acceptable range of 98–102%. The accuracy and precision level (RSD%) for the 5 replicates prepared at LOQ level, 10× LOQ, and a concentration in between proved to be at least 97.5 and ≤2%, respectively, as shown in Table 2.

**Table 2.** Precision, accuracy, and sample fortification data for DFZ (EC 10%).

Nominal concentration	0.004 µg ml^−1^	0.01 µg ml^−1^	0.04 µg ml^−1^
Mean	0.00388	0.0094	0.0395
Standard deviation	0.00004	0.000135	0.00017
^*a*^RSD% (precision)	1.030	1.39	0.45
Accuracy %	97	97.4	98.8
*N* (replicate number)	5	5	5

This table shows the analysis of five samples at three fortification levels of DFZ 10% EC which are the LOQ (0.004 µg ml^−1^) and 10-fold the LOQ (0.4 µg ml^−1^) and a concentration in between (0.01 µg ml^−1^).

^*a*^RSD% is the percentage of RSD among samples of each tested concentration level.

### Validation of the accurate wash-off volume

All plant leaves of the crops mentioned in Table 1 were treated with the same concentration of DFZ EC 10% (0.625 mg ml^−1^), showing different Aerosol OT 0.01% volumes were needed to reach a complete removal of the residue from the leaf surface. A different number of washes and different volumes of the wash-off solution [Aerosol OT 0.01% (w/v)] were needed to rinse off all the DFZ residue from the leaf surfaces even though all of these plants have been sprayed with the same concentration of the DFZ EC 10%.

Dwarf French bean leaves needed 30 ml Aerosol OT 0.01% (w/v) in one wash to dislodge all the pesticide from the leaf surface. Similarly, tomato leaves needed a total of 30 ml of the wash-off solution but in two consecutive washes of the same leaves. Wheat leaves, oilseed rape, and soya bean needed 45 ml of the same wash-off solution to reach the lower quantification limit of the sprayed DFZ 10% EC as illustrated in Table 3. The total surface area of the treated leaves (double sided) has been estimated using the millimetre graph paper method, and values are recorded in Table 1.

**Table 3.** Validation of the wash-off solution (Aerosol OT 0.01%) volume needed to dislodge DFZ EC 10% on different plant leaves.

Crops/variety	Number and olume of washes	Washing interval (min)	Wash concentration mean (±SD) (µg ml^−1^)	RSD% per wash
Dwarf French bean ‘*Phaseolus vulgaris*’ Variety: Tendergreen	First wash (30 ml)	15 min	0.022 (±0.003)	14.4%
	Second wash (15 ml)	15 min	<LOQ^*a*^	N/a
Wheat leaves ‘*Triticum aestivum*’ Variety: Skyfall	First wash (30 ml)	15 min	0.166 (±0.010)	6%
	Second wash (15 ml)	15 min	0.017 (±0.002)	11.7%
	Third wash (15 ml)	15 min	˂LOQ^*a*^	N/a
Oilseed rape ‘*Brassica napus*’ Variety: Charger	First wash (15 ml)	15 min	0.029 (±0.004)	12.2%
	Second wash (15 ml)	15 min	0.024 (±0.004)	14.4%
	Third wash (15 ml)	15 min	0.021 (±0.003)	14.7%
	Fourth wash (15 ml)	15 min	<LOQ^*a*^	N/a
Tomato ‘*Solanum lycopersicum*’ Variety: Alicante	First wash (30 ml)	15min	0.10 (±0.009)	8.5%
	Second wash (15 ml)	15 min	0.013 (±0.001)	9.5%
	Third wash (15 ml)	15 min	<LOQ^*a*^	N/a
Soybean ‘*Glycine max*’ Variety: Green Shell	First wash (15 ml)	15 min	0.119 (±0.022)	18.3%
	Second wash (15 ml)	15 min	0.044 (±0.004)	9.6%
	Third wash (15 ml)	15 min	0.024 (±0.003)	12.3%
	Fourth wash (15 ml)	15 min	<LOQ^*a*^	N/a

This table illustrates the validation of different volumes of the wash-off solution needed for different plants when sprayed with DFZ 10% EC. SD ± mean is the standard deviation of *n* = −5 determinations. RSD% is the percentage of RSD between replicates of each wash. N/a (not applicable): the data did not allow the calculation due to the initial value below LOQ and not being detectable.

^*a*^Values below the LOQ ≤ 0.004 were considered zero.

### Exemplar of DFZ EC 10% DFR estimation using the laboratory introduced method

The actual amount of DFZ in the initial spray deposited was 5.2 µg due to a slight increase in the concentration prepared (0.651 mg ml^−1^) compared with the targeted concentration (0.625 mg ml^−1^). The targeted concentration was derived from the typical application rate of DFZ used in the field (125 g per 200 l per ha). From the validation of the accurate wash-off volume needed, oilseed rape leaves needed 45 ml of Aerosol 0.01% (w/v) in three consecutive washes (15 ml each) for all the dislodgeable residue to be removed.

The DFR data gathered from the treatment proved to be normally distributed (*P* = 0.592) when tested using the Shapiro–Wilk test using SPSS, IBM version 27.0 (IBM, 2020) The normality of DFR data generated in the current study is unlike what has generally been reported as the distribution of the DFR data in the open literature (Korpalski *et al.*, 2005). The newly developed laboratory method indicated the DFR residue on the oilseed rape leaves is approximately 34.2% of the initial amount deposited which corresponds to an average of 1.78 µg of DFZ while the control group indicated no detected DFR residue as illustrated in Table 4.

**Table 4.** DFR of DFZ 10% EC on oilseed rape leaves.

Treatment	Amount recovered in each 15 ml wash volume (µg) Mean (±SD)			Total amount of DFZ recovered (µg) Mean (±SD)	Variance across washes (RSD%)^*a*^	DFR recovery (%) Mean (±SD)	Shapiro–Wilk normality test (*P* value) (Significance *P* = 0.05)
	First wash	Second wash	Third wash				
Treated group	0.95 (±0.19)	0.49 (±0.09)	0.33 (±0.06)	1.78 (±0.34)	19%	34.2% (±6.6%)	0.59
Control group	−0.001 (±0.00) <LOQ^*b*^	−0.002 (±0.00) <LOQ^*b*^	0.000 (±0.00) <LOQ^*b*^	N/a	N/a	0	N/a

This table elucidates the DFR of DFZ 10% EC 3 h after spraying on oilseed rape leaves. Data represent the mean (±SD) of *n* = 10 determinations. SD ± mean is the standard deviation. DFZ DFR residue on oilseed rape plants proved to be normally distributed when tested by the Shapiro–Wilk test (*P* = 0.59) using SPSS*. N/a (not applicable): the data did not allow the calculation due to the initial value below LOQ and not being detectable.

^*a*^RSD% is the percentage of RSD among samples of each tested concentration level.

^*b*^Values below the LOQ ≤ 0.004 were considered zero.

By testing the method for the dislodgeability of DFZ EC 10% on oilseed rape the opportunity to interpret the DFR value in the field is provided, presuming the similarity of the environmental conditions in both sites. This can be calculated by considering the application rate (125 g DFZ/200 l per ha) and the average surface area of the treated leaves to evaluate the DFR in µg Active substance cm^−2^ kg^−1^ ha^−1^. For instance, the average leaves’ surface area in the experiment was 132 cm^2^ (66 cm^2^ per double-sided leaf) thus, by normalizing the recovered residue (1.78 µg) with the application rate in kgs (0.125) and dividing it with the estimated surface area, an estimation of the DFR value could expected to be approximately of 0.10 µg DFZ cm^2^ kg H^−1^. The DFR value resulted from this lab experiment is 30-folds less than the default value of DFR set by EFSA (3 µg).

## Discussion

The DFR analytical method implemented proved to be accurate and precise following the OECDE/GD(97)184 guidance for the conduct of studies of occupational exposure to pesticide during agriculture application (OECD, 1997). While the guidelines recommended an accuracy value between 70 and 120%, the method accuracy was between 98 and 102%. For the analytical laboratory’s capability to perform accurate and precise analysis, the precision value less than or equal to 20% (RSD) is recommended. However, the current precision of the developed method is less than or equal to 2%. The acceptable mean recovery range is known to be between 90 and 110% for formulations of a 10% concentration according to the European Commission guidance for generating and reporting method of analysis of PPPs (European Commission, 2019). However, the mean recovery of each sample solution of the five fortification levels of DFZ EC 10% exceeded the above-mentioned level indicating the suitability of the method especially since the method has been applied in the laboratory under relatively controlled conditions compared with field studies. Thus, maintaining a higher precision and accuracy was recommended for quality assurance.

Moreover, the precision and accuracy of the three fortification levels tested have been confirmed to comply with the quality assurance criteria of the SANTE/2020/12830 Rev.1 methods (European Commission, 2021) indicating a suitable range of precision and accuracy used in the analysis.

DFR data generated from the newly developed tested laboratory method proved to be normally distributed (*P* = 0.59) using the Shapiro–Wilk test. However, detected normality is uncommon for such kind of residue in the field studies as it is well known to follow the log-normal distribution (Korpalski *et al.*, 2005). However, Korpalski *et al.* (2005) study was only on a very small scale using two pesticides (i.e. carbaryl and methomyl) and only one crop (i.e. cabbage) using the wash-off technique described in the initial DFR method first developed by Gunther *et al.* (1973). This study did not validate the amount of wash-off needed for each pesticide to completely dislodge available residues from the surface and hence this could increase the variability among the data gathered. The reason for such normality achieved in the laboratory-developed study when tested on oilseed rape could be due to the high precision and accuracy (±2%) used in the method as described above along with validating the wash-off solution volume needed to rinse off all the residue from the leaf surface. This helped in achieving homogeneity of the residue level detected per wash. Another reason for normality could be the uniform growth of the tested plants and operating the experiment under constant environmental conditions which could be hard to achieve in the DFR field studies.

The sample dislodging procedures described in the DFR current methodology involves washing the whole leaves or leaf punches of a specific surface area (400 cm^2^ double sided) in two aliquots of aqueous surfactant solution [i.e. Aerosol OT (0.01%, w/v)], shaking the leaves in the aqueous surfactant solution for 10 min before retaining the residue for the analysis (EPA, 2009). Nevertheless, the guidelines of the method indicated the need for a further technique to confirm the efficacy of the washing-off solution to rinse all the residue from the treated leaves (EPA, 2009). Unlike the field experiment, in the lab experiment, sampling the whole leaf was favourable to capture all the pesticide deposits and reduce the variability that could exist due to selecting leaf portions that are not equally treated. In addition, some leaves are not applicable to be sampled by leaf punchers (i.e. wheat).

From the data generated in this study, different leaves or crops proved to require different wash-off solution volumes to rinse all the pesticide residue from the leaf surface as shown in Table 3. Dislodging all leaves in the same volume of the wash-off solution regardless of the existing differences between them could underestimate the dislodged fraction of the pesticide leading to misleading or at least inconsistent quantification of the DFR and consequently poor comparison between different DFR estimations. Such a gap in the literature could lead to an inaccurate estimation of the non-dietary risk associated with the use of PPPs.

For further illustration of the importance of such proof-of-concept study, a simple calculation of the amount of wash-off solution being used in the current DFR methodology and most GLP studies suggests 1 cm^2^ of leaf surface area would need 0.5 ml of the washing of solution. This estimation is based on the fact that the method recommends using 200 ml (two equal aliquots) of the wash-off solution to rinse 400 cm^2^ of leaves surface area. By applying the same calculation on the tested leaves (i.e. dwarf French bean, wheat, oilseed rape, tomato, and soya bean) with their required volume of the wash-off solution proved in the proof-of-concept study above, apart from oilseed rape which would require 0.3 ml from the washing-off solution, all the other crops/leaves required more than 0.5 ml which is currently used in the method (whilst acknowledging that the method reported here was validated for whole leaves rather than punched leaf discs which are typically used in field studies). This illustrated the importance of validation step in quantifying the full dislodgeable fraction of the pesticide residue left on the surface after the spray dries.

Data on the DFR of pesticide are extremely rare in the literature (Badawy *et al.*, 2021) and this has been recognized by the EFSA in their guidance on assessing the exposure of operators, workers, residents, and bystanders in risk assessment for PPPs (EFSA, 2014a; Charistou *et al.*, 2022). The report clearly stated the need to generate and collect further DFR data to reflect a realistic default value. Moreover, the public consultation on the same guidance also emphasized the lack of data correlation between factors that affect DFR and the DFR values. Such a gap in the literature could be due to the high cost of the field DFR studies and as such any generated data remain confidential and only used for registration purposes.

Studying these factors that could be influencing the degree of pesticide dislodgeability could be a key solution in facilitating the registration and allow extrapolation between studies based on a scientific finding from the laboratory-developed method described above. Especially, this technique provides a high level of analytical accuracy and precision along with more confidence using the validation above to properly estimate the DFR relative to the applied dose. This laboratory-developed method could also be a step in identifying the expected residue and decline in the field before commencing more extensive and expensive field studies. The research carried out in the laboratory will potentially inform further research under field conditions to identify factors that could affect DFR using different formulations, adjuvants, meteorological conditions, and many other factors solely or in combination.

The current study was a proof-of-concept study to investigate the suitability of a controlled laboratory method to estimate DFR values. Using this laboratory method paves the way for other research to systematically study factors that may influence DFR such as different leaf types, temperature, humidity, formulations, and co-formulas. The outcomes from such research could be vital in generating data that allow multiple comparisons with the in-field data generated by the industry to support the registration of PPPs. It should be noted that some in-field applications and spraying methods could be challenging to correlate with the future generated data from this method such as those driven from the drone/aerial spray or the ultra-low volume sprays. Nevertheless, studying the factors that may affect DFR also at ultra-low volume or aerial spray (i.e. leaves, formulations, etc.) using our new laboratory DFR method could help in estimating a correlation factor to predict and extrapolate between the in-field generated data if applicable. Such research would also in turn, add to the data available for consideration alongside those currently available to draw a better conclusion on the DFR and its influencing factors.

## Conclusion

The newly developed DFR laboratory method has been assessed and shown to be a fast, easy, and cost-effective method to predict the dislodgeable residue on plant leaves. The observations from this laboratory work could be transcribed to field conditions and in the future when field data become available to a degree that allows robust statistical analysis, comparisons could be made between the two. The method is also deemed to be controllable and can be managed and operated in different desirable environmental conditions or seasons that best describe the DFR field conditions. Besides, the described validation of the method adds certainty to the generated data and allows better prediction of the dislodgeable residue level. In conclusion, this method could facilitate studying different factors that could affect the degree of DFR saving time and resources for the regulatory authorities and the agrochemical industry.

## Supplementary Material

wxac045_suppl_Supplementary_Material

## Data Availability

The authors confirm that the data supporting the findings of this study are available within the article.
