# Supplementary material for: Development of a New Dislodgeable Foliar Residue Analytical Laboratory Method for Pesticides
Source: Ann Work Expo Health. 2022 Jun 29;66(8):1070–80. doi: 10.1093/annweh/wxac045 (PMC10483580; doi:10.1093/annweh/wxac045)
Supplement: wxac045_suppl_Supplementary_Material [file wxac045_suppl_supplementary_material.docx]

**Title:** **Development of a new dislodgeable foliar residue analytical laboratory method for pesticides.**

Authors: Mohamed H. Badawy, Darragh Murnane, Kathleen A Lewis, and Neil Morgan

**Supplemental Materials**

| **Nominal Conc. (µg mL^-1^)** | **Average Response**  **Area** | **Conc. Average**  **(µg mL^-1^)**  **SD (+/-)** | ***Precision (RSD%)** | ***Accuracy%** |
| --- | --- | --- | --- | --- |
| 0.004 | 9801.8 | 0.0039  (± 0.00002) | 0.57% | 102.0% |
| 0.005 | 12117.4 | 0.004916  (± 0.00006) | 1.38% | 101.7% |
| 0.006 | 14647 | 0.0060  (± 0.00004) | 0.68% | 99.9% |
| 0.008 | 19298 | 0.0080  (± 0.00006) | 0.78% | 99.9% |
| 0.01 | 24413.8 | 0.01021  (± 0.00007) | 0.75% | 102.1% |
| 0.02 | 46951 | 0.01994  (± 0.00005) | 0.27% | 99.7% |
| 0.04 | 93515 | 0.03998  (± 0.00012) | 0.04% | 99.9% |

**Table S1: Precision and accuracy calculation of the concentration gradient used in the difenoconazole analysis**

*Table S1 shows the calculated precision and accuracy for the concentration gradient used in the difenoconazole analysis.*

** RSD% is Relative Standard Deviation %.*

**STDV is Sample Standard Deviation.*

** Accuracy is calculated by the division of the theoretical concentration by the prepared concentration and multiplied by 100.*
